# Bioinspired Design of Alcohol Dehydrogenase@nano TiO_2_ Microreactors for Sustainable Cycling of NAD^+^/NADH Coenzyme

**DOI:** 10.3390/nano8020127

**Published:** 2018-02-24

**Authors:** Sen Lin, Shiyong Sun, Ke Wang, Kexuan Shen, Biaobiao Ma, Yuquan Ren, Xiaoyu Fan

**Affiliations:** 1 Key Laboratory of Solid Waste Treatment and Resource Recycle of Ministry of Education, Institute of Non-Metallic Minerals, Southwest University of Science and Technology, Mianyang 621010, China; linsenzxc@163.com (S.L.); wangke066@126.com (K.W.); skx179az@163.com (K.S.); qinqinmabiao@163.com (B.M.); renyuquan0839@163.com (Y.R.); 2 Low-cost Wastewater Treatment Technology International Sci-Tech Cooperation Base of Sichuan Province, Mianyang 621010, China; 15929421073@163.com

**Keywords:** enzyme encapsulation, bioinspired microreactors, nicotinamide coenzymes cycling, alcohol dehydrogenase, TiO_2_ nanoparticles

## Abstract

The bioinspired design and construction of enzyme@capsule microreactors with specific cell-like functionality has generated tremendous interest in recent years. Inspired by their fascinating complexity, scientists have endeavored to understand the essential aspects of a natural cell and create biomimicking microreactors so as to immobilize enzymes within the hierarchical structure of a microcapsule. In this study, simultaneous encapsulation of alcohol dehydrogenase (ADH) was achieved during the preparation of microcapsules by the Pickering emulsion method using amphiphilic modified TiO_2_ nanoparticles (NPs) as building blocks for assembling the photocatalytic microcapsule membrane. The ADH@TiO_2_ NP microreactors exhibited dual catalytic functions, i.e., spatially confined enzymatic catalysis and the membrane-associated photocatalytic oxidation under visible light. The sustainable cycling of nicotinamide adenine dinucleotide (NAD) coenzyme between NADH and NAD^+^ was realized by enzymatic regeneration of NADH from NAD^+^ reduction, and was provided in a form that enabled further photocatalytic oxidation to NAD^+^ under visible light. This bioinspired ADH@TiO_2_ NP microreactor allowed the linking of a semiconductor mineral-based inorganic photosystem to enzymatic reactions. This is a first step toward the realization of sustainable biological cycling of NAD^+^/NADH coenzyme in synthetic functional microsystems operating under visible light irradiation.

## 1. Introduction

In recent years, the design and construction of enzyme@capsule microsystems inspired from the fundamental functions of subcellular organelles has attracted significant attention [1,2,3]. The development of such highly compartmentalized enzymatic microreactors is motivated by a wide range of applications, such as transformation of energy [4,5,6,7], understanding the origin of protolife [8,9,10], and production of bioactive species with membrane-bounded microcompartments [1,3,11,12].

One of the critical components of bioinspired enzyme@capsule microreactors is the microcapsule membrane employed for the encapsulation or immobilization of the enzyme [8,13,14]. One of the most frequently used techniques for the preparation of a microcapsule is the Pickering emulsion approach, by which solid particles are assembled at the oil/water interface to produce stable emulsion droplets. To date, a great number of diverse microcapsule membranes with hierarchical structures have been developed from individual building blocks [8,13,14,15]. Recent studies have shown that inorganic microcapsules with a core-shell structure can be prepared from amphiphilic modified nanoparticles (NPs) of amorphous silica [16], mesoporous silica [17], Fe_3_O_4_ [9], TiO_2_ [18], TiO_2_/Fe_3_O_4_ hybrid, or clay particles [8,19].

**Figure 1:**
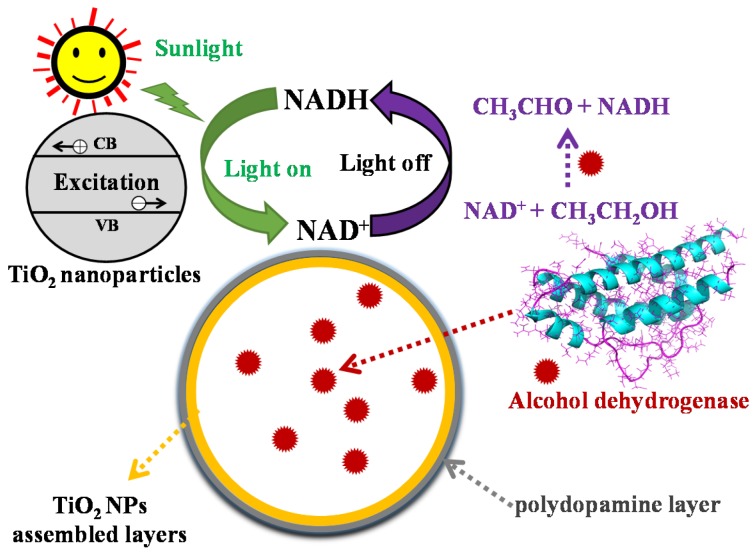
Schematic illustration of the cycling of nicotinamide coenzymes NAD^+^/NADH catalyzed by the bioinspired ADH@TiO_2_ NP microreactors. The polydopamine layer was formed by means of in situ polymerization of dopamine and used as a photosensitizer for the activation of the TiO_2_ NP membrane under visible light. During cycling of NAD^+^/NADH, enzymatic regeneration of NADH from NAD^+^ reduction is catalyzed by alcohol dehydrogenase and subsequent photocatalytic oxidation to NAD^+^ takes place by the PDA-modified TiO_2_ NP membrane under visible light irradiation.

Nicotinamide adenine dinucleotide (NAD) is a coenzyme found in all living cells that drives a number of enzyme-catalyzed reactions. It exists in two forms, an oxidized and a reduced form, which are abbreviated as NAD^+^ and NADH, respectively [5,20]. Although several reports have addressed the design and construction of artificial microreactors for mimicking the behavior of cellular regeneration or oxidation of NADH, there are relatively few investigations concerning the sustainable cycling of the NAD^+^/NADH coenzyme. Notably, TiO_2_ NPs, as efficient semiconducting NPs, have often been used in different forms, e.g., as a free aqueous dispersion [21], nanocomposite [22], or a confined dispersion in microcapsules [5,9,23]. Herein, we have described bioinspired photoresponsive microreactors comprised of encapsulated alcohol dehydrogenase (ADH) and TiO_2_ NP-assembled microcapsule membranes (Figure 1). Our objective was to initiate the key steps towards sustainable coenzyme cycling based on micro-compartmentalized ADH@TiO_2_ NP microreactors. Consequently, the photoactive membrane and the enzyme confined within the synthetic cell-like microcapsule were integrated together. In this regard, amphiphilic modified TiO_2_ NPs were used as the Pickering stabilizer for the preparation of water-in-oil colloidosomes (microcapsules). ADH was simultaneously encapsulated into the TiO_2_ NP-stabilized microcapsules during the preparation of water-in-oil Pickering emulsion droplets. The as-prepared water-in-water ADH@TiO_2_ NP microreactors were modified with a layer of polydopamine (PDA) to enhance the utilization of visible light. PDA was formed by the polymerization of dopamine (DA) after being cross-linked and transferred into the aqueous phase. Specifically, we initiated the spatially confined enzyme reaction within a population of ADH@TiO_2_ NP microreactors to generate NADH from NAD^+^ reduction, which diffused through the medium and induced subsequent membrane-associated photocatalytic oxidation to produce NAD^+^ under visible light irradiation. During the reduction process, the ethanol in the reaction system lost a hydrogen atom under the catalytic action of ADH within the microreactors, and subsequently, the NAD^+^ gained this free hydrogen atom and was reduced to NADH. Under visible light irradiation, NADH was catalytically oxidized to NAD by the PDA-modified TiO_2_ NP membrane on the surface of the microreactors. Subsequently, the oxidized NAD^+^ regenerated NADH again in the dehydrogenation reaction catalyzed by the ADH and newly added ethanol. Thus, the sustainable cycling of NAD^+^/NADH coenzyme was realized by using ADH@TiO_2_ NP microreactors.

## 2. Materials and Methods

### 2.1. Materials

Commercial 3-aminopropyltriethoxysilane (APTES)-modified TiO_2_ NPs (TiO_2_ content ≥ 95%) were purchased from Huizhe Fine Chemistry Co., Ltd. (Suzhou, China). Hexamethylene tetramine (HMTA, purity ≥ 99.5%) and hydrogen peroxide (purity 30%) were obtained from Aladdin Biochemical Technology Co., Ltd. (Shanghai, China). DA (purity ≥ 98%), NAD^+^ (purity ≥ 98%), NADH (purity ≥ 98%), Fluorescein isothiocyanate (FITC) and ADH were purchased from Hefei Bomei Biological Technology Co., Ltd. (Hefei, China). Ethanol, toluene, tetraethoxysilane (TEOS) and Tris-HCl were purchased from Chengdu Kelong Chemical Reagent Factory (Chengdu, China). All other chemicals were of analytical grade and used without further purification. All water used in experiments was obtained from Millipore water purification system Milli-Q Integral 5 (Paris, France) (resistance ≥ 18.25 MΩ·cm^−1^).

### 2.2. Preparation of PDA-Modified ADH@TiO_2_ NP Microreactors

The ADH@TiO_2_ NP water-in-oil microcapsules (microreactors) were prepared by the Pickering emulsion method [8]. A calculated amount of TiO_2_ (10 mg) was dispersed in 10 mL of toluene and sonicated in an ultrasonic bath for 15 min. Typically, an aqueous solution containing 200 μL of ADH or FITC-labeled ADH (FITC-ADH) (100 U·mL^−1^) was added to 2 mL toluene containing 1 mg·mL^−1^ TiO_2_ dispersion. The mixture was homogenized for 1 min using a homogenizer (F10, Fluko, Berlin, Germany) at 10,000 rpm. Subsequently, the surface of the water-in-oil microcapsules was cross-linked by hydrolytic reaction with 70 µL of TEOS for 24 h. The water-in-water ADH@TiO_2_ NP microreactors were prepared after toluene was removed by ethanol washing and transferred into the continuous water phase.

The surface of the ADH@TiO_2_ NP microreactors was modified with PDA, which was formed by the polymerization of dopamine under alkaline conditions, in order to improve their photocatalytic activity [19]. The ADH@TiO_2_ NP microreactors were incubated with 2 mL of 0.2 mg·mL^−1^ DA aqueous solution. Then, 4 mg of HMTA was added to the suspension to induce the formation of the PDA layer on the surface of the microcapsules under the weakly alkaline environment. The PDA-coated ADH@TiO_2_ NP microreactors were isolated by centrifugation of the reaction mixture at 8000 rpm for 5 min. The ADH@TiO_2_ NP microreactors were washed thrice with ethanol.

### 2.3. NAD^+^/NADH Cycling Catalyzed by ADH@TiO_2_ NP Microreactors

First, 1 mM NAD^+^ and 1 mM NADH solutions were prepared in a buffer (100 mM Tris-HCl and 10 mM sodium phosphate, pH 7.5). In a typical NADH generation procedure, 300 μL of the NAD^+^ solution and 1.6 mL of the buffer solution were added into 0.01 mg·mL^−1^ dispersion of the ADH@TiO_2_ NP microreactors. Then, 100 μL of ethanol (concentration was 50%) was added to the mixture to initiate the enzymatic reaction at 37 °C. The concentration of generated NADH was determined by monitoring the increasing intensity of the UV-visible absorption band at 340 nm at given time intervals using a micro-spectrophotometer with an integrated normal cuvette (Nanodrop 2000C, Thermo, Shanghai, China). For the oxidation of NADH, the reaction mixture was placed into a quartz reactor equipped with a stirring bar. The visible light irradiation source was a 300 W Xenon lamp (AuLight CEL-HXF300, Beijing, China) with a cut-off filter of 420 nm. The visible light intensity in the photocatalytic test section was 1300 mW·cm^−2^. The distance between the reactor and the light lamp was fixed at 5 cm. During the experiment, the concentration of NAD^+^ was estimated by measuring the decreasing absorption at 340 nm. Finally, another 100 μL of ethanol was added to the reaction system to initiate the regeneration of NADH at 37 °C without light illumination. The cycling of NAD^+^/NADH was evaluated more than 20 times.

### 2.4. Characterization

The morphology of the prepared microcapsules was observed by an optical microscope (DM 2000, Leica, Bonn, Germany), scanning electron microscope (SEM, Ultra 55, Zeiss, Berlin, German), and transmission electron microscope (TEM, Libra 200, Zeiss, Berlin, Germany). The microreactors were also characterized by Fourier transform infrared (FT-IR) spectroscopy using a Spectrum One (American PE, Cincinnati, OH, USA) spectrometer. The UV-Vis absorption spectra were recorded on an ultraviolet solid diffuse reflectometer (UV-DRS, UV-3150, Shimadzu, Kyoto, Japan). The particle size distribution of the tourmaline particles was characterized by a particle size analyzer (Plus 90, Brookhaven, Holtsville, NY, USA). The mineral phase of tourmaline was characterized by an X-ray diffractometer (X’pert Pro, PANalytical, Eindhoven, The Netherlands). Confocal laser scanning microscopy (CLSM) images of the distributions of FITC-labeled ADH within the microcapsules were obtained on an inverted CLSM (TCS SP8, Leica, Mannheim, Germany).

## 3. Results and Discussion

**Figure 2:**
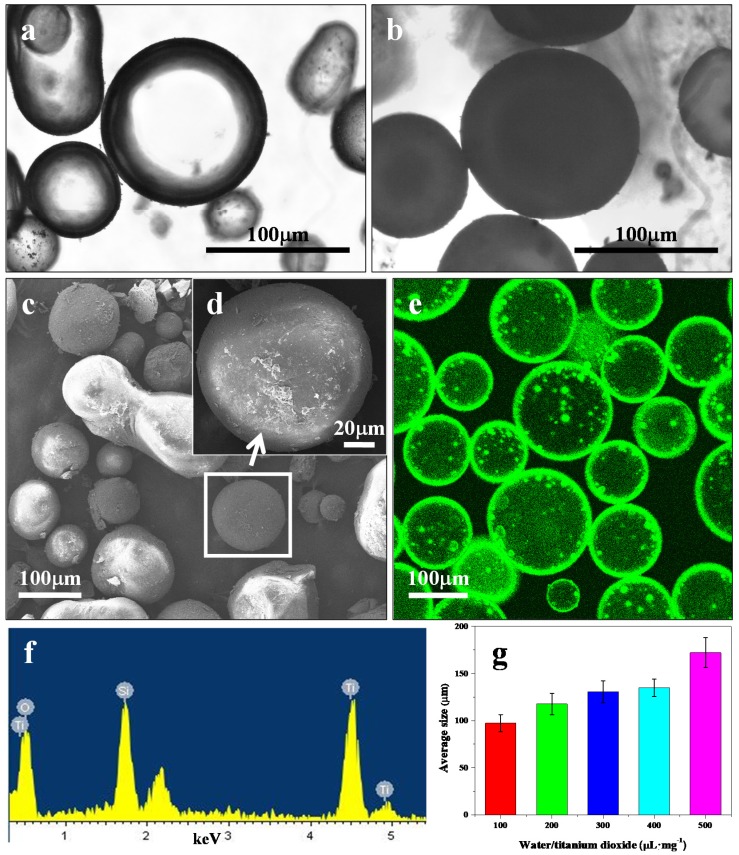
Microscopic images of the ADH@TiO_2_ NP microreactors. (**a**,**b**) Optical images; (**c**,**d**) SEM images; and (**e**) CLSM image. (**a**) Water-in-oil Pickering emulsions before being cross-linked by TEOS; (**b**) Water-in-water microcapsules after being cross-linked by TEOS; (**c**,**d**) SEM images of the dried ADH@TiO_2_ NP microreactors; (**e**) CLSM image of the FITC labeled ADH@TiO_2_ NP microreactors; (**f**) EDS analysis of the ADH@TiO_2_ NP microreactors; (**g**) Average size distributions, median values, and standard deviations were calculated by fitting Gaussian functions to the histograms.

The water-in-oil Pickering emulsion microcapsules were prepared by using amphiphilic TiO_2_ NPs as a stabilizer at the water-toluene interface. APTES is an aminosilane that is frequently used in the process of amphiphilic surface modification of nanoparticles. Therefore, APTES-grafted TiO_2_ NPs were used in the experiments as the Pickering stabilizer. The APTES-modified TiO_2_ NPs were in anatase phase with an average diameter of ~74 nm and contact angle of ~83.7° (see details in Figures S1–S4, Supporting Information). The resulting water-in-oil emulsion microcapsules were prepared after the TiO_2_ NPs assembled at the oil-water interface. Optical microscopy images showed discrete spherical microcapsules (Figure 2a and Figure S5, Supporting Information). The microcapsule membrane was cross-linked by the TEOS hydrolysis reaction to stabilize the structural integrity after being transferred to the aqueous phase (Figure 2b) [8,19]. SEM images showed that the dried microcapsules had distinctive TiO_2_ NP layers with rough surfaces Figure 2c,d,f. ADH was simultaneously encapsulated into the TiO_2_ NPs microcapsules and assembled into ADH@TiO_2_ NP microreactors. CLSM observations of the fluorescent FITC-labeled membranes confirmed that ADH was well-encapsulated into the TiO_2_ NP membranes (Figure 2e). The ADH@TiO_2_ NP microreactors exhibited size polydispersity, which was dependent on the water/TiO_2_ volume/weight ratio used in the preparations. The mean diameter varied from ~60 to 110 μm with ratios ranging from 50 to 250 µL·mg^−1^ (Figure 2g and Figure S6, Supporting Information).

TiO_2_ is a well-known n-type semiconductor that has long been used for photocatalysis [5,21,22,23]. However, pristine TiO_2_ NPs with a band gap larger than 3.0 eV are only active under ultra-violet light illumination [24]. To utilize a wider solar spectrum of visible light and enable visible light photoactivation of pristine TiO_2_ NPs, modification with PDA is an effective strategy, and is achieved by the in situ polymerization of dopamine onto the surface of the microcapsules [24]. Therefore, PDA surface modification of ADH@TiO_2_ NP microreactors was also performed in our study. The pristine TiO_2_ NPs show absorption only in the UV region without any detectable absorption of visible light (Figure S6, Supporting Information). In contrast, the UV-Vis adsorption spectrum of PDA-modified ADH@TiO_2_ NP microreactors indicated significant visible light absorption, suggesting that the solar energy conversion efficiency was enhanced after PDA surface modification (Figure S6, Supporting Information). FT-IR spectra of the ADH@TiO_2_ NP microreactors showed characteristic peaks of TiO_2_ NPs, ADH, and PDA, suggesting the coexistence of encapsulated ADH, PDA, and TiO_2_ NP microcapsule membranes (Figure S7, Supporting Information).

**Figure 3:**
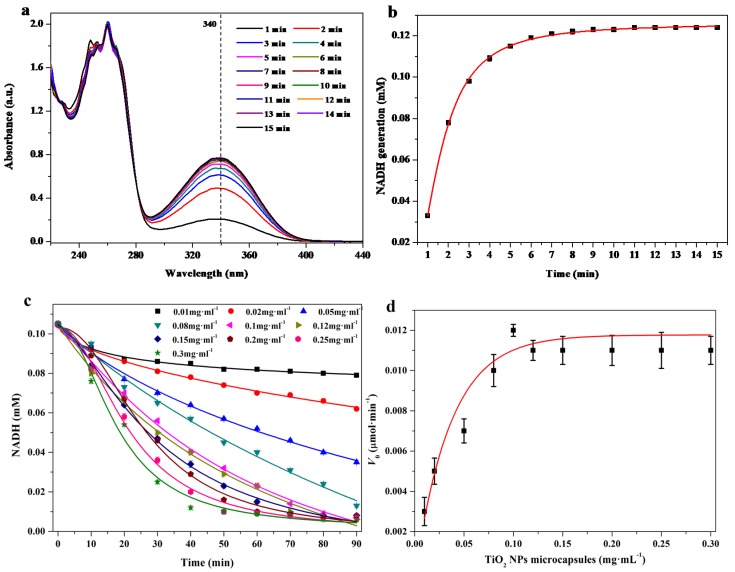
UV-Vis spectra showing that the increase in absorption intensity at 340 nm is consistent with the reduction of NAD^+^ to NADH in a reaction mixture containing NAD^+^ (1 mM, 300 µL), ethanol (50%, 100 µL), and ADH (10 µg·mL^−1^, 100 µL) at 37 °C and pH 7.5. (**a**) Corresponding plots of intensity at 340 nm vs. time; (**b**) Plots of NADH concentration vs. time. UV-Vis absorption intensity at 340 nm was consistent with the oxidation of NADH to NAD^+^ under visible light irradiation in a reaction mixture containing NADH (1 mM, 200 µL), TiO_2_ NP microcapsules without ADH and with varying TiO_2_ NP microcapsules concentrations from 0.01 to 0.3 mg·mL^−1^ at 37 °C and pH 7.5; (**c**) Corresponding plots of NADH concentration decreasing vs. time; (**d**) Initial velocity (*V*_0_) vs. concentration of the microcapsules.

The enzymatic activity of free ADH and visible light-driven photocatalysis of TiO_2_ NPs microcapsules without ADH were separately evaluated. The reaction dispersions were exposed to either a 5000 lux visible light source or placed in the dark with the addition of ethanol. The concentration of NADH was calculated by the absorption intensity of its characteristic peak at 340 nm (Figure S8, Supporting Information) [18]. The formation of NADH was catalyzed by free ADH and monitored by the increase in the absorption at 340 nm Figure 3a,b. Conversely, the generation of NAD^+^ from the oxidation of NADH under visible light in the reaction mixture was estimated by the decreasing intensity of NADH Figure 3c,d. Normally, both free ADH and TiO_2_ NP microcapsules without ADH presented their own catalytic performances Figure 3.

**Figure 4:**
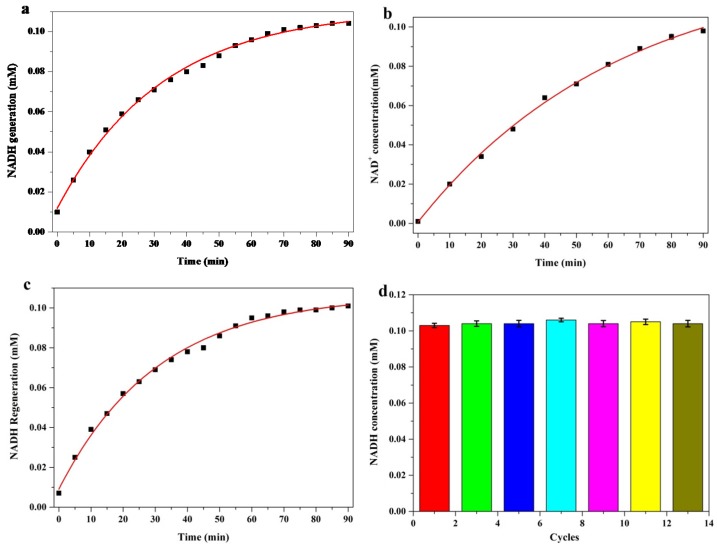
NAD^+^/NADH cycling performance catalyzed by ADH@TiO_2_ NP microreactors. (**a**) Reduction of NAD^+^ to NADH; (**b**) Oxidation of NADH to NAD^+^ under visible light irradiation; (**c**) Regeneration of NADH from oxidized NAD^+^; (**d**) Cycling performance in 13 cycles. A reduction reaction mixture containing ADH@TiO_2_ NP microreactors (0.1 mg·mL^−1^), NAD^+^ (1 mM, 300 µL), and ethanol (50%, 100 µL) at 37 °C and pH 7.5.

The cycling performance of NAD^+^ and NADH catalyzed by ADH@TiO_2_ NP microreactors is shown in Figure 4. In the first step of a typical cycling of NADH generation-NADH oxidation to NAD^+^-NADH regeneration, NAD^+^ was catalytically reduced to NADH by the spatially confined ADH. In the second step of cycling, NADH oxidation in reaction system was initialized upon exposure to visible light irradiation (Figure 4b). To complete the cycle, NADH was regenerated by the addition of ethanol to switch on the ADH function for the reduction of NAD^+^ to NADH without visible light illumination (Figure 4c). According to the experimental results (Figure 4a–c), sustainable biological cycling of NAD^+^/NADH coenzyme was realized in the synthetic functional microreactors under visible light irradiation. The catalytic stability of the ADH@TiO_2_ NP microreactors was also investigated (Figure 4d). The results indicated that the ADH@TiO_2_ NP microreactors were sufficiently stable even after 13 consecutive cycles.

## 4. Conclusions

In summary, novel bioinspired photoresponsive ADH@TiO_2_ NP microreactors were developed to facilitate the mimicking of sustainable nicotinamide coenzymes of NAD^+^/NADH cycling in a synthetic functional microcompartment under visible light irradiation. The designed microreactors represent a model of a simple nonbiological microsystem that is related to enzymatic catalysis and inorganic photochemical reactions. Thus, our research provides new opportunities in visible light energy capture and transduction. Furthermore, it is possible to synthesize this simple microsystem easily and on a large scale. Consequently, the proposed membrane-bounded microcompartments with core-shell hierarchical structure have great potential in photoactive microreactor design and drug delivery.

## Supplementary Materials

The following are available online at http://www.mdpi.com/2079-4991/8/2/127/s1, Figure S1: X-ray diffraction pattern of 3-aminopropyltriethoxysilane (APTES) modified TiO_2_ NPs, Figure S2: Transmission electron microscope (TEM) characterization of APTES modified TiO_2_ NPs. TEM images (a,b), Figure S3: Size distribution of APTES modified TiO_2_ NPs. The average particle size is ca. 74 nm, Figure S4: Photographs of water droplets mounted on the surface of TiO_2_ NPs showing stable droplets on APTES modified TiO_2_ NPs due to amphiphilic character (a); and droplet spreading on conventional hydrophilic P25-type TiO_2_ NPs (b), respectively. The corresponding mean contact angle for droplets on APTES modified TiO_2_ NPs was ca. 83.7°, Figure S5: Size distribution of ADH@TiO_2_ NPs microreactors with water/TiO_2_ volume/weight ratio from 50 µL·mg^−1^ (a); 100 µL·mg^−1^ (b); 150 µL·mg^−1^ (c); 200 µL·mg^−1^ (d) and 250 µL·mg^–1^ (e), Figure S6: UV-Vis adsorption spectral characterization of TiO_2_ NPs, PDA and PDA modified ADH@TiO2 NPs microreactors with varied concentrations from 0.1 to 1 mg·mL^−1^, Figure S7: FTIR spectral characterization of TiO_2_ NPs, PDA and PDA modified ADH@TiO_2_ NPs microreactors with varied concentrations from 0.1 to 0.6 mg·mL^−1^, Figure S8: Chemical formula (a) and UV-Vis spectra (b) of NAD^+^ and NADH.
