# Supplementary material for: Bioinspired Design of Alcohol Dehydrogenase@nano TiO2 Microreactors for Sustainable Cycling of NAD+/NADH Coenzyme
Source: Nanomaterials (Basel). 2018 Feb 24;8(2):127. doi: 10.3390/nano8020127 (PMC5853758; doi:10.3390/nano8020127)
Supplement: Supplementary file 1 [file nanomaterials-08-00127-s001.docx]

**Supporting information**

**Bioinspired Design of Alcohol Dehydrogenase@nano TiO_2_ Microreactors for Sustainable Cycling of NAD^+^/NADH Coenzyme**

Sen Lin ^1, 2^, Shiyong Sun ^1, 2*^, Ke Wang ^1, 2^, Kexuan Shen ^1^, Biaobiao Ma ^1^, Yuquan Ren^1^, Xiaoyu Fan^2^

^1^ Institute of Non-metallic Minerals, Key Laboratory of Solid Waste Treatment and Resource Recycle of Ministry of Education, Southwest University of Science and Technology, Mianyang 621010, China. linsenzxc@163.com(S. L.); wangke066@126.com(K. W.); skx179az@163.com(K. S.); qinqinmabiao@163.com(B. M.); renyuquan0839@163.com(Y. R.); 15929421073@163.com (X. F.).

^2^ Low-cost Wastewater Treatment Technology International Sci-Tech Cooperation Base of Sichuan Province, Mianyang 621010, China

***** Correspondence: shysun@swust.edu.cn; Tel.: +86-816-2419569

**Figure S1.** X-ray diffraction pattern of 3-aminopropyltriethoxysilane (APTES) modified TiO_2_ NPs.


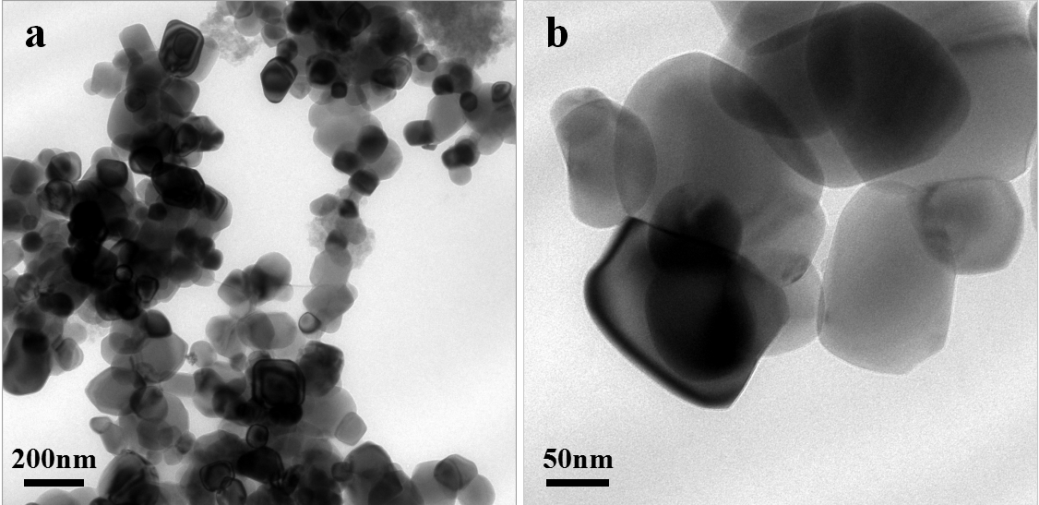


**Figure S2.** Transmission electron microscope (TEM) characterization of APTES modified TiO_2_ NPs. TEM images (**a**, **b**).

**Figure S3.** Size distribution of APTES modified TiO_2_ NPs. The average particle size is
ca. 74 nm.


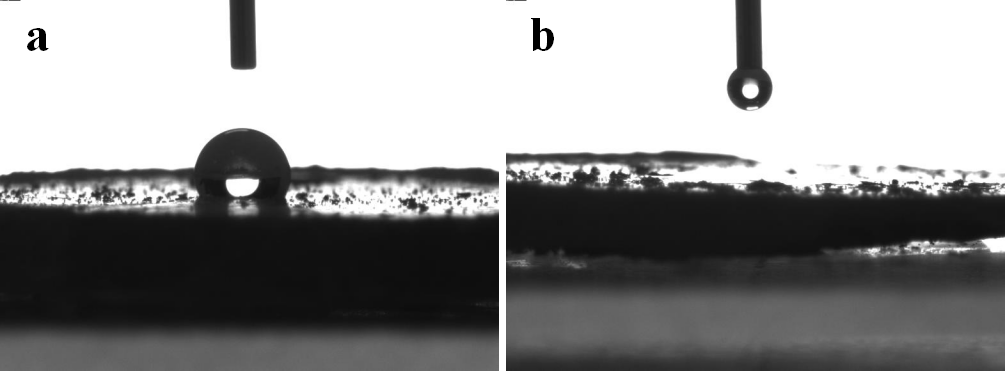


**Figure S4.** Photographs of water droplets mounted on the surface of TiO_2_ NPs showing stable droplets on APTES modified TiO_2_ NPs due to amphiphilic character (**a**); and droplet spreading on conventional hydrophilic P25-type TiO_2_ NPs (**b**), respectively. The corresponding mean contact angle for droplets on APTES modified TiO_2_ NPs was ca. 83.7^o^.

**Figure S5.** Size distribution of ADH@TiO_2_ NPs microreactors with water/TiO_2_ volume/weight ratio from 50 µL·mg^-1^ (**a**); 100 µL·mg^-1^ (**b**); 150 µL·mg^-1^ (**c**); 200 µL·mg^-1^ (**d**) and 250 µL·mg^−1^ (**e**).

**Figure S6.** UV-vis adsorption spectral characterization of TiO_2_ NPs, PDA and PDA modified ADH@TiO_2_ NPs microreactors with varied concentrations from 0.1 to 1 mg·mL^−1^.

**Figure S7.** FTIR spectral characterization of TiO_2_ NPs, PDA and PDA modified ADH@TiO_2_ NPs microreactors with varied concentrations from 0.1 to 0.6 mg·mL^-1^.




**Figure S8.** Chemical formula (**a**) and UV-Vis spectra (**b**) of NAD^+^ and NADH.
